# Supplementary material for: Comparing demographics of signatories to public letters on diversity in the mathematical sciences
Source: PLoS One. 2020 Apr 28;15(4):e0232075. doi: 10.1371/journal.pone.0232075 (PMC7188238; doi:10.1371/journal.pone.0232075)
Supplement: S1 Appendix — Here we provide the full text of Letters A, B, and C, as well as the full text of our Amazon Mechanical Turk questionnaire. (PDF) [file pone.0232075.s001.pdf]

Supporting Information

Chad M. Topaz [1,2*], James Cart[3], Carrie Diaz Eaton[4], Prisca Gayles[5], Jude A. Higdon[2,6], Kenan Ince[7], Brian Katz[8], Drew Lewis[9], Jessica Libertini[10], Alicia Prieto Langarica[11]

1 Department of Mathematics and Statistics, Williams College, Williamstown, MA, USA
2 Institute for the Quantitative Study of Inclusion, Diversity, and Equity, Williamstown, MA, USA
3 Office of Institutional Research, Williams College, Williamstown, MA, USA
4 School for Interdisciplinary Studies, Bates College, Lewston, ME, USA
5 Department of Africana Studies, Williams College, Williamstown, MA, USA
6 Office of Information Technology, Bennington College, Bennington, VT, USA
7 Department of Mathematics, Westminster College, Salt Lake City, UT, USA
8 Department of Mathematics, Augustana College, Rock Island, IL, USA
9 Department of Mathematics and Statistics, University of South Alabama, Mobile, AL, USA
10 Department of Applied Mathematics, Virginia Military Institute, Lexington, VA, USA
11 Department of Mathematics and Statistics, Youngstown State University, Youngstown, OH, USA

* qside@qsideinstitute.org

S1 Appendix: QSIDE Post 1

QSIDE brings quantitative expertise together with expertise from the social sciences, humanities, and arts to discover the impact and scope of injustices and to build solutions to remedy them. When those injustices are themselves within the world of quantitative expertise, it hits painfully close to home.

What happened

The most widely-read mathematics publication in the world is Notices of the American Mathematical Society. The most recent issue of the Notices contains an essay by Prof. Abigail Thompson, who is both a Vice President of the American Mathematical Society and Chair of the Department of Mathematics at UC Davis. The essay speaks against the use of diversity statements in college and university faculty hiring, and equates the practice to McCarthyism. Really read the piece yourself, if you like. QSIDE prefers not to re-hash it here. What is a diversity statement?

In a typical faculty search process, candidates submit a cover letter, a cv, a research statement (describing their research experience and future plans) and a teaching statement (describing their teaching experience and philosophy). Additionally, many faculty searches now require candidates to submit a diversity statement. The purpose of the statement is to identify candidates who have skills, experiences, and/or plans that would support inclusion, diversity, and equity on campus. This is reasonable. Some people will equate the use of a diversity statement with "politics" or "affirmative action." Not so. A straight white cisgender man can write a stupendously effective diversity statement if he learns about the issues and thinks about how to address them in his professional life. And as for politics, diversity statements are not politics, at least, not in the partisan sense. They are policy. Some institutions of learning, to their credit, have decided that they would like to address inclusion, diversity, and equity issues. Seeking faculty who can teach in inclusive ways, for example, is a perfectly reasonable job requirement, just as we expect to hire faculty who can perform research.

What is going on at the American Mathematical Society?

Why would the Notices choose to publish Thompson's piece? The opening sentence, "This essay contains my opinions as an individual," is no consolation. The Notices has a choice in what it publishes,

and it uses its editorial discretion all the time. By amplifying Thompson's views to a large audience, the American Mathematical Society lends legitimacy to them, and in doing so, engages in both-sides-ism. It's ok to use diversity statements, on one hand, and diversity statements are like McCarthyism, on the other hand. This raises the unavoidable question: AMS, what are your own actual views on diversity statements?

What is going on at UC Davis?

The Department of Mathematics at UC Davis is, in many ways, not diverse. Recognizing the problems and limitations of assessing demographics based on names and pictures, we nonetheless have done so because we do not have any other available data on the department. We have limited ourselves here to some quick gender statistics. We infer the tenure-stream faculty in the department to be 18% women. While this is dreadfully low, it is also right around the national average. That said, the national distribution does have spread, so there are many departments doing much better than UC Davis. The situation with graduate students in much more concerning. According to AMS statistics, at the national level, women comprise just shy of 30% of Ph.D. recipients in the mathematical sciences. As for UC Davis' Department of Mathematics, we infer the pool of graduate students to be 14% or 15% women. Department leadership and/or culture being hostile to the idea of asking faculty candidates to articulate diversity and inclusion plans could certainly be linked to the department's abysmal gender representation. This raises questions for UC Davis: Are you concerned about the diversity issues in the Department of Mathematics? Are you concerned that you have a department chair who publicly opposes your institution's requirement of using diversity statements? Regardless of her personal views, have you now looked into whether she is abiding by your institution's rules around these statements?

What can you do in response

Here is a list I have come up with so far. I will edit this post as more ideas surface.

1. Email AMS Executive Directory Catherine Roberts (exdir@ams.org), AMS President Jill Pipher (jill_pipher@brown.edu), and Notices Editor-in-Chief Erica Flapan (ELF04747@pomona.edu). You are welcome to use this text I wrote:

   > Dear AMS,
   >
   > I am truly dismayed by the AMS's decision to publish Abigail Thompson's piece in the December Notices. You have given a far-reaching platform to dangerous views that build a false equivalency between diversity and inclusion, on one hand, and on the other hand, McCarthyism.
   >
   > The "personal opinion" disclaimer at the start of the essay is no consolation. Your editorial choices carry weight and they convey values. Why did you devote page space to Thompson's piece? Do you find it legitimate? What are you own views around the use of diversity statements in hiring? If I asked to publish a personal opinion piece that said "Women are better mathematicians than men" would you let me?
   >
   > I believe you have made a grave and very damaging mistake by publishing Thompson's essay.
   >
   > Respectfully, Chad

2. Stop doing favors for the Notices. Full disclosure: QSIDE already has one piece that will soon appear in the Notices and it is past the point where we have the capability of pulling it. However, I was asked to write another piece for the Notices a few months down the road and I have now written them to cancel my anticipated submission. Please consider not working with the Notices until they reckon with their decision to publish Thompson's piece.

3. Spread the word about this debacle on social media and in your workplaces.

4. Contact UC Davis, Thompson's institution, to express your concerns about diversity in the Department of Mathematics and about Thompson's role as Chair. If she has gone on record in a very public way as being opposed to diversity statements, and if UC Davis requires them, the school must look into whether or not she has been abiding by institutional policy.

5. For those of you who are in mathematics, advise grad-school-bound undergraduate students especially students who are minoritized along some axis not to apply to UC Davis. Advise your graduate student and postdoc colleagues not to apply there for jobs. I can already hear some people saying "if we are advising minorities not to go there, how will their abysmal diversity situation ever get better?" In response, I would say that it is not the job of minoritized people to go into bad situations in order to diversify them. The department and the university must lead, and must demonstrate proactively that they have created policies and practices that promote a healthy and inclusive environment.

6. Please donate to QSIDE. We are a 501(c)3 tax-exempt nonprofit organization and we need your support. Every single penny you donate goes towards quantitative work on social justice issues.

S2 Appendix: Letter A

We are a group of concerned mathematicians writing in response to AMS Vice President Abigail Thompson's editorial, invited by the AMS for publication in the December 2019 edition of the Notices. In this editorial, Dr. Thompson states her personal opinion against the mandated use of faculty diversity statements in hiring decisions and compares such requirements to McCarthyist loyalty oaths.

We are all members of many mathematical societies, including the American Mathematical Society. Some of us serve on committees in these societies or are chairs of committees in these societies. Some of us are or have been chairs of departments, some of us are or have been chairs of search committees, and some of us have written or reviewed diversity statements as part of search processes. We have all thought deeply about the role of diversity statements and related tools, such as student success statements.

We are compelled to write because the AMS leadership's actions have harmed the mathematics community, particularly mathematicians from marginalized backgrounds. We are writing because we support diversity statements as one tool to encourage a more inclusive and equitable mathematics profession. We are writing because we wish to correct the misleading impressions readers might have of such statements from Thompson's editorial: Thompson's opinion does not represent the opinions of many other members of the mathematics community. We are writing because not everyone is in a position to raise their voice. We are writing because it matters how our community and its leaders talk about diversity, especially in our profession's most prominent publication. We are writing because we are disappointed by the editorial decision to publish the piece which contradicts the AMS's commitment to diversity affirmed in its own diversity statement. Clearly, this is something that people needed to talk about, but the AMS has chosen to spark this conversation by giving its imprimatur to a piece that undermines productive discussion and causes real danger and burden to the marginalized members of our community.

Diversity statements are widely used in academic hiring as one component to assess candidates' qualifications for the job. Each statement one requires as part of a hiring process research, teaching, mentoring, service, or diversity helps paint a picture of how a candidate will contribute to the work of an institution. Increased use of diversity statements reflects a growing recognition in higher education that faculty contribute in positive ways to the campus community by acknowledging, appreciating, and collaborating with groups of students, staff, and fellow faculty who are diverse along varied axes. In acknowledgement that this is part of the work of a faculty member and of the hiring process, we recommend that graduate programs explicitly prepare their graduates to contribute to this work and to write and talk about it meaningfully, and we commend the programs already undertaking this work. There are plenty of legitimate questions about how to use diversity statements effectively and how (more broadly)

to create diverse and supportive faculties. In order to reduce bias in the evaluation of candidates, hiring committees evaluate statements according to criteria that indicate evidence of these important contributions, grounded in the missions of higher education in general and their institution in particular. Asking for and evaluating diversity statements are not quick solutions to the complex challenge of justice and inclusion in higher education, but they can help hiring committees to evaluate candidates' skills in doing this portion of our professional work.

Diversity statements help assess a candidate's ability to effectively teach a diverse group of students. If our goal as mathematicians and educators is truly to reach as many students as possible, thinking about diversity and inclusion is necessary. Good teaching is necessarily inclusive. If we willfully ignore an important area of pedagogy that demonstrably helps more students succeed in math, then we will continue to reproduce systems of inequity, and we will do a great disservice to our students. We will therefore not be effective teachers.

Suggesting that actively attempting to include more students in mathematics is equivalent to the Red Scare is ignorant (about both history and the present) and dangerous. Claims of "reverse racism," which equate critiquing privilege with oppressing the privileged, have a long and unsavory history in and beyond higher education. Without understanding the history in which these discussions are rooted, it is possible to profess support for the ideal of equality while acting in ways that lead to exclusion and inequity.

While Dr. Thompson attempts to spin this issue with partisan wording, diversity statements are a small yet necessary step towards creating a more equitable and inclusive community. Higher education in the US is shifting, student populations we serve are changing, and our understanding of how to better serve all students is advancing. We need a rehumanization of mathematics that can affirm students' cultural funds of knowledge while examining and combating its own roles in supporting power structures. We need leadership at all levels, from professional societies to presidents, boards, deans, and chairs, to recognize this reality, advocate for students and faculty from a variety of backgrounds, and move us forward.

Dr. Thompson's preface that the letter is her "personal opinion" does not alleviate our concerns, nor does the fact that she seems to be referring primarily to the use of these documents at the UC system. The fact remains that the Notices made an editorial decision to give Thompson's essay a national (indeed, international) platform, and in a prominent position within the publication. Notices is a publication of the AMS, and Dr. Thompson is identified as an AMS officer in her byline. According to Notices editor Erica Flapan, Dr. Thompson's position in the AMS leadership led the AMS to solicit her letter. These contextual details send a message to the profession about how diversity is viewed by those with power and responsibility in the AMS and a major university department. The AMS and Notices bear responsibility for amplifying views that fly in the face of research-based practices and that falsely equate evidence-based approaches to teaching and professional practice with the blacklisting of people based on political ideology, all in direct contradiction of the AMS's stated commitment to diversity.

AMS's own diversity statement claims, "The American Mathematical Society is committed to promoting and facilitating equity, diversity and inclusion throughout the mathematical sciences We reaffirm the pledge in the AMS Mission Statement to 'advance the status of the profession of mathematics, encouraging and facilitating full participation of all individuals,' and urge all members to conduct their professional activities with this goal in mind." While merely publishing Dr. Thompson's letter demonstrates the AMS's lack of commitment to this statement, the fact that it was written by and credited to an officer of the AMS raises even more serious questions about the statement's sincerity.

We strongly disagree with the sentiments and arguments in Dr. Thompson's editorial, and we hope that the AMS will reconsider the way that it uses its power and position in the mathematics communities in these kinds of discussions. However, we primarily write this letter to our fellow mathematicians and students of all kinds who might have wondered if inclusion work is valued in our community. We want students and faculty, especially those with multiple identities that are minoritized in mathematics, to know that many mathematicians see this inclusion work as integral to our community and identity.

S3 Appendix: Letter B

We write with grave concerns about recent attempts to intimidate a voice within our mathematical community. Abigail Thompson published an opinion piece in the December issue of the Notices of the American Mathematical Society. She explained her support for efforts within our community to further diversity, and then described her concerns with the rigid rubrics used to evaluate diversity statements in the hiring processes of the University of California system.

The reaction to the article has been swift and vehement. An article posted at the site QSIDE urges faculty to direct their students not to attend and not to apply for jobs at the University of California, Davis, where Prof. Thompson is chair of the math department. It recommends contacting the university to question whether Prof. Thompson is fit to be chair. And it recommends refusing to do work for the Notices of the American Mathematical Society for allowing this piece to be published.

Regardless of where anyone stands on the issue of whether diversity statements are a fair or effective means to further diversity aims, we should agree that this attempt to silence opinions is damaging to the profession. This is a direct attempt to destroy Prof. Thompson's career and to punish her department. It is an attempt to intimidate the AMS into publishing only articles that hew to a very specific point of view. If we allow ourselves to be intimidated into avoiding discussion of how best to achieve diversity, we undermine our attempts to achieve it.

We the undersigned urge the American Mathematical Society to stand by the principle that important issues should be openly discussed in a respectful manner, and to make a clear statement that bullying and intimidation have no place in our community.

S4 Appendix: Letter C

In an essay in the December 2019 issue of the Notices, Abigail Thompson describes the mandatory "Diversity Statement" (mDS) that mathematics job applicants to UC Schools must submit together with their regular applications. At some campuses, the mDS is evaluated, in various categories, according to a detailed list of criteria (called a "rubric").

If an applicant to Berkeley, for example, merely says that she advocates mentoring, treating all students the same regardless of background, she merits a score of 1–2 out of a possible top score of 5 in the track record for advancing diversity category (see * below). Hiring committees (at UC Davis and Berkeley, in particular), are encouraged by the Administration to use the rubrics, establish a cutoff and eliminate candidates who score below the cutoff as a first step in the hiring process for all hires. In this way Diversity Statements diminish the value of mathematical achievement as the key element in securing a position at a UC Mathematics Department.

Mandatory Diversity Statements undermine Faculty Governance. Should the use of scored diversity statements become required as the first step in the hiring process, this opens the way for Administrators, who have no professional knowledge of mathematics, to exert primary control over the hiring of mathematicians. And indeed, testing the waters, small scale pilot programs have already been implemented at various UC schools requiring the first cut on hiring to be based on such scored diversity statements.

We applaud Abigail Thompson for her courageous leadership in bringing this issue to the attention of the broader Mathematics Community. As she says in her essay:

"Mathematics has made progress over the past decades towards becoming a more welcoming, inclusive discipline. We should continue to do all we can to reduce barriers to participation in this most beautiful of fields.... There are reasonable means to further this goal: encouraging students from all backgrounds to enter the mathematics pipeline, trying to ensure that talented mathematicians don't leave the profession, creating family-friendly policies, and sup-porting junior faculty at the beginning of their careers, for example."

We agree wholeheartedly with these sentiments. It is important to strive to hire faculty that will

make the atmosphere more welcoming to all. It is also important to recognize and help reduce various difficulties still faced by underrepresented groups. But as Abigail says, there are mistakes to avoid: mDSs are one of them.

Finally, we commend the Editorial Board at the Notices for opening up the discussion on this very important matter.

* If you insert the following into a google search, the first thing which comes up is a pdf with the rubric for Berkeley: rubric_to_assess_candidate_contributions_to_diversity_equity_and_inclusion-1.pdf

S5 Appendix: Survey Instrument

We are interested in gathering demographic information about people who signed a high profile public letter to an academic/professional society. Immediately below, you will see the way a person signed the letter.

[public signature text information]

We ask you to find certain demographic information about the person above. To find this information, you might use:

- Wikipedia
- Google searches
- The Mathematics Genealogy Project
- any other resources you find helpful

1. The information above needs to be separated into a person's name and professional affiliation. Please enter the person's name as they have written it above. That is, separate the person's name from all other information, and enter it here. If there are typos or extra spaces, please clean these up.

   [free text entry]

2. The information above needs to be separated into a person's name and professional affiliation. Please enter the person's professional affiliation. That is, separate the affiliation from all other information and enter it here. This should be the name of an educational institution or a company ONLY. If the person has given other information (for instance, title, academic department, professional specialization, honors/awards, random geographic info that is not part of the company's or academic institution's name) please ignore it in answering this question. If the person has listed multiple affiliations, enter only the first one. If the person has not listed an affiliation (just a name) then see if you can locate the person's affiliation (by Googling) and enter it here. If you cannot determine their affiliation with confidence, please leave this question blank.

   [free text entry]

3. Please try to locate web information associated with the person. This might be their own personazlied [sic] web site and/or the web site of a department, college/university, or company. Please enter at most one website, even if you use more in answering other questions below. If you cannot locate any web information, please leave this question blank.

   [free text entry]

4. Please try to locate an email address associated with the person. A professional email address (college/university, company) is preferred. If you cannot locate an email address, please leave this question blank.

   [free text entry]

5. What do you think the person's gender is? In answering this question, you might use their name, and/or any pictures you find of them, and/or any textual information you find referring to them with gendered pronouns.

   - Woman
   - Man
   - Binary man/woman choice does not apply (nonbinary, gender nonconconforming)
   - Cannot confidently determine (insufficient time/information)

6. An ethnic group is a category of people who identify with each other based on similarities such as common ancestral, linguistic, social, cultural or national experiences. What is the person's primary ethnic background?

   - American Indian or Alaska Native. A person having origins in any of the original peoples of North and South America (including Central America), and who maintains tribal affiliation or community attachment.
   - Black or African American. A person having origins in any of the black racial groups of Africa.
   - Asian. A person having origins in any of the original peoples of the Far East, Southeast Asia, or the Indian subcontinent including, for example, Cambodia, China, India, Japan, Korea, Malaysia, Pakistan, the Philippine Islands, Thailand, and Vietnam.
   - Latinx. A person of Cuban, Mexican, Puerto Rican, South or Central American origin, or similar.
   - Native Hawaiian or Other Pacific Islander. A person having origins in any of the original peoples of Hawaii, Guam, Samoa, or other Pacific Islands.
   - Middle East or North Africa. A person having origins in locations such as Algeria, Morocco, Egypt, Lebanon, or Syria.
   - White. A person having origins in Europe.
   - Other (not listed above).
   - Cannot confidently determine (insufficient time/information).

7. Based on the person's affiliation as given above (and, as necessary, based on other information you find) does the person have an affiliation with higher education (student or employee at a college, university, or other post-secondary educational institution)? Note: please answer yes for a person who is retired or "emeritus" from teaching in higher education.

   - Yes, the person is affiliated with higher education.
   - No, the person is not affiliated with higher education.
   - Cannot confidently determine (insufficient time/information).

   If the answer to question 7 above is NO or CANNOT DETERMINE, please scroll down to the bottom of this form and submit it without answering any further questions. If the answer to question 7 is YES, please continue.

8. We would like to know a little bit more about the person's college/university. In particular we'd like to know if it is in the United States, and if so, its degree of research intensiveness. As for research intensiveness, please see this Wikipedia page. A college/university may be in the first list on that page as "R1: Doctoral Universities Very high research activity", or in the second list on that page as "R2: Doctoral Universities High research activity" or on neither list. Choose the appropriate option below.
    - The institution is in the United States and is on the R1 list.
    - The institution is in the United States and is on the R2 list.
    - The institution is in the United States but is on neither the R1 list nor the R2 list.
    - The institution is not in the United States.
    - Cannot confidently determine (insufficient time/information).
9. If you selected NOT IN THE UNITED STATES in question 8 above, please type the full name of the country where the institution is located. Otherwise, please leave this question blank.

    [free text entry]
10. We would like to know a little bit more about the person's role/title at their college/university. Choose the appropriate option below.
    - The person is a retired professor (they may or may not have the title Emeritus).
    - Their title is Professor (or an international equivalent).
    - Their title is Associate Professor (or an international equivalent).
    - Their title is Assistant Professor (or an international equivalent).
    - The person is an instructor who is not eligible for academic tenure. For instance, their title is Instructor or Lecturer or Postdoctoral Fellow, or it contains one of the following phrases: "NTT", "non-tenure track," "visiting", or similar.
    - The person is a graduate student (earning master's degree or PhD).
    - The person is an undergraduate student (earning bachelor's degree or similar).
    - The person is staff at the institution (they are not a student nor are they any of the faculty types listed above).
    - Other (none of the choices above).
    - Cannot confidently determine (insufficient time/information).
11. If you chose STAFF, OTHER, or CANNOT DETERMINE for question 10, please skip this question. Otherwise, we would like to know a little bit more about the person's academic field. This might be based on their title, academic department, degree program, or other information. Choose the appropriate option below.
    - Mathematics or Applied Mathematics.
    - Statistics or Data Science.
    - Computer Science, Computer Engineering, or similar.
    - Mathematics Education (this means that the person's research and/or studies pertain to how best to teach mathematics).
    - Other (none of the choices above).
    - Cannot confidently determine (insufficient time/information).

12. If in question 10 you chose GRADUATE STUDENT, UNDERGRADUATE STUDENT, STAFF, OTHER, or CANNOT DETERMINE, please skip this question. Otherwise, for question 8, you indicated one of the first five choices (some type of professor or other instructor). Determine the year that person earned a PhD degree. You might find this on their web page, their curriculum vitae, or in the Mathematics Genealogy Project. Please enter the Ph.D. year as a four-digit number. If you cannot find this information, or if you believe the person does not have a Ph.D., please leave this question blank.

    [free text entry]
